# Concurrent injection of dexamethasone intravitreal implant and anti-angiogenic agent in patients with macular edema: A retrospective cohort study

**DOI:** 10.1097/MD.0000000000008868

**Published:** 2017-11-27

**Authors:** Hung-Yu Lin, Chia-Yi Lee, Jing-Yang Huang, Shun-Fa Yang, Shih-Chun Chao

**Affiliations:** a Department of Ophthalmology, Show Chwan Memorial Hospital, Changhua; b Institute of Medicine; c Department of Optometry, Chung Shan Medical University, Taichung; d Department of Optometry, Yuanpei University of Medical Technology, Hsinchu; e Department of Medical Research, Chung Shan Medical University Hospital, Taichung; f Department of Electrical and Computer Engineering, National Chiao Tung University, Hsinchu; g Department of Optometry, Central Taiwan University of Science and Technology, Taichung, Taiwan.

**Keywords:** anti-angiogenic, dexamethasone, intraocular pressure, macular edema, Ozurdex

## Abstract

To evaluate the safety and efficiency in macular edema patients who concurrently received a single injection of a dexamethasone intravitreal implant (DEX, 0.7 mg) and ranibizumab (2.3 mg).

A retrospective cohort study was conducted, and medical records from 2012 to 2016 were reviewed. Patients who received concurrent DEX and ranibizumab injections with a follow-up period of at least 3 months were enrolled in the study group. An age and gender-matched group received ranibizumab injections and was designated the control group. The best-corrected visual acuity (BCVA), central macular thickness (CMT) and intraocular pressure (IOP) were included in the analysis. Steroid-induced ocular hypertension (SIOH) is defined as either an elevation of more than 10 mmHg from baseline or a single IOP measurement of more than 30 mmHg.

A total of 26 patients were enrolled in the current study with 13 patients in each group. Both the BCVA (*P* = .04) and CMT (*P* < .01) achieved significant improvement after the follow-up period in the study group. The IOP increased after the injection but no significant elevation was observed throughout the follow-up period in the study group (*P* = .15). For SIOH, 1 patient in the study group had an elevated IOP of 10 mmHg (7.7%) at 2 postoperative months, and no single IOP measurement of more than 30 mmHg was obtained. Five patients (38.5%) in the study group received medical treatment that successfully retarded their IOP elevation, and no individuals required surgical management. In the control group, there were no significant fluctuations concerning BCVA, CMT, and IOP, and no ocular hypertension was observed. According to the inter-group analysis, the CMT and BCVA recovered more significantly in the study group than in the control group.

Concurrent injection of DEX and ranibizumab is a preliminary method that shows effectiveness in treating ME. Furthermore, safety is also guaranteed, with moderate levels of severity and transient IOP elevation being observed. A future large-scale study is necessary to evaluate the long-term effects and safety of this combined treatment.

## 1 Introduction

The macula accounts for the majority of visual function, and damage to the macula contributes to various visual symptoms, such as metamorphopsia, photopsia, decreased color vision, lower daytime visual acuity and blind spot formation. Macular edema (ME), which is related to fluid retention in the macular regions, increases macular thickness beyond its normal value, which is usually approximately 260 μm.^[1]^ The etiology of ME commonly results from inflammation or ischemic changes, which damage visual performance.^[2]^

Corticosteroids were first used for human diseases in 1948.^[3]^ Since then, they have been widely used to treat inflammatory and edematous disorders on the ocular surfaces and retina, including ME, to improve visual acuity and retard edematous status.^[3,4]^ However, significantly elevated intraocular pressures (IOP) after topical corticosteroid therapy have been reported since 1962,^[5]^ and several subsequent studies have also revealed a vision-threatening complication known as steroid-induced ocular hypertension (SIOH).^[3,6,7]^ A patient's predisposing factors, as well as the dose and potency of steroids influence the development of SIOH; among other topical steroids, topical dexamethasone has the strongest potency and greatest ability to raise IOP.^[8]^ Ocular hypertension (OHT) is a major risk factor for open angle glaucoma, which can cause legal blindness,^[9]^ therefore, different types and routes of corticosteroid therapy should be carefully evaluated.

Dexamethasone intravitreal implant (DEX, brand name: Ozurdex; Allergan, Inc., Irvine) is an intravitreal bioerodible, sustained-release device that was first approved to treat ME in 2009.^[10]^ DEX can be controlled-released from the device within 6 months, with gradual delivery of medication after the polymer device undergoes hydrolysis in the vitreous cavity.^[11,12]^ Currently, DEX is used to manage the ME induced by branch retinal venous occlusion, central retinal venous occlusion, posterior uveitis and diabetic macular edema.^[11,13]^ It shows similar effectiveness compared to anti-angiogenic agents but with less frequent injections.^[14]^ Nevertheless, SIOH has been reported in patients who received DEX implantations,^[12–16]^ and the degree and management of SIOH after DEX implantation is uncertain. After DEX implantation, the extent of SIOH ranges from transient elevations^[17]^ to a severe form, for which medication and even surgical management could be warranted in some patients.^[15,18]^ Furthermore, only 1 study has revealed similar effects between combined DEX and anti-angiogenic therapy and ranibizumab monotherapy, which demonstrate similar outcomes, while multiple injections of the combined therapy did not show an advantage over monotherapy, considering the convenience and cost.^[19]^

Our study aimed to evaluate the efficiency and safety of concurrent DEX and anti-angiogenic agent injection in terms of the changes to visual performance, retardation of macular thickness, and IOP elevation in patients with macular edema. Medications used to retard SIOH and complications (other than SIOH) will also be discussed.

## 2 Materials and methods

All procedures and managements performed in this study involving human participants adhered to the 1964 Declaration of Helsinki and its later amendments. The present study was also approved by the Institutional Review Board at Show Chwan Memorial Hospital (Registration No. 1060502).

A retrospective cohort study was conducted at Show Chwan Memorial Hospital. Medical records were reviewed in detail, and patients who received concurrent DEX and ranibizumab injections from 2012 to 2016, with a follow-up period of at least 3 months, were eligible for study enrollment. The following exclusion criteria were applied patients who had undergone previous glaucoma surgery, long-term systemic steroid users, patients with neovascular glaucoma, visual acuity worse than hand motion at any distance, and patients who received previous treatment for ME before the current management. An age and gender-matched group who had received ranibizumab (Lucentis, Novartis, Basle, Switzerland) injections 3 times over a 3-month period from 2012 to 2016 was selected as the control group.

All DEX implantations were performed by a single retinal specialist (HYL) using the same surgical steps. Patients were informed of the risks and possible complications, including increases to IOP and glaucoma occurrence. The surgery was performed with the patients under local anesthesia. After sterilization, DEX 0.7 mg (Ozurdex; Allergan, Inc., Irvine) was injected into the vitreous cavity, 3.5 to 4.0 mm posterior to the limbus using an injector provided by the company. Ranibizumab 2.3 mg (10 mg/mL) was injected intraoperatively after DEX implantation. After the completion of the injection, an anterior chamber paracentesis was performed. Oral acetazolamide and topical gentamicin were applied after the injection, and a pressure patch was used to seal the wound. In the visits following the combined injection, each patient's IOP status was evaluated, and patients with elevated IOP more than 6 mmHg were first treated with travoprost (Travatan, Alcon, TX) or brimonidine (Alphagan-p; Allergan, Inc), as determined by the glaucoma specialist (S.C. Chao). If an IOP elevation of more than 15 mmHg or an IOP of more than 35 mmHg was observed for more than 1 month, despite medical treatment, then surgical intervention was considered.

Patient characters, including basic data, systemic diseases and ocular disorders, were investigated. Primary ophthalmic outcomes, including best-corrected visual acuity (BCVA), central macular thickness (CMT) and IOP fluctuations, were measured approximately 1 week before the surgery, 2 months after the surgery and 3 months after the surgery. The BCVA was measured with a Snellen chart at a 6-m distance. The CMT was obtained using an optical coherence tomography (PRIMUS 200; Carl Zeiss, Co., Ltd, Jena, Germany) image. The CMT area in this study is based on the macular thickness map, while the average thickness of the central 5 subfields enclosing the fovea were included in the following analysis. The IOP was measured through pneumatic tonometry (NT-2000; Nidek Co., Ltd, Gamagori, Japan), with 3 measurements for all patients; the averaged value was used in the following analysis if the difference among the 3 measurements did not exceed 3 mmHg. A prominent SIOH was defined as either an IOP elevation of more than 10 mmHg from baseline or a single IOP measurement of more than 30 mmHg during the follow-up period. The secondary outcomes included postoperative complications and adverse effects, such as recurrent vitreous hemorrhage, retinal detachment, corneal edema, cataract formation, etc.

SPSS version 20.0 (SPSS Inc., Chicago, IL) was used for all statistical analyses in this study. The visual acuity value was transformed into a logarithm of the minimal angle of resolution (LogMAR) for analysis. In the standard set by Holladay et al^[20]^ and the University of Freiburg's study group results,^[21]^ hand movement was defined at 0.005/2.3 (decimal/logMAR), and counting fingers was defined at 0.014/1.85, and these values were also used in this study. Chi-squared test was performed to analyze differences in the basic data, including sex, laterality, and major disease. The Kruskal–Wallis test was used in the intra-group analysis, while the Mann-Whitney *U* test was used to compare the differences between the study and control groups. If the result of Kruskal–Wallis test was significant, post-hoc pairwise comparison would be conducted. A *P* value of <.05 was considered to be statistically significant, and a confidential interval of 95% was declared. Furthermore, *P* values of less than 0.01 were depicted as *P* < .01. The statistic power is approximately 0.78 under the 0.05 alpha value and medium effect size using G∗power version 3.1.9.2 (Heinrich-Heine-Universität, Düsseldorf, Germany).

## 3 Results

A total of 13 patients (mean age of 57.30 ± 12.02 years) were enrolled in the study group, and another 13 patients (mean age of 61.08 ± 7.15 years) were included in the control group. There were 8 males and 5 females in both groups, while the laterality showed 8 right eye lesions and 5 left eye lesions in the study group and 7 right lesions and 6 left lesions in the control group. The indication of treatment included 8 individuals with retinal venous occlusion (RVO) in the study group and 5 individuals with RVO in the control group. One patient in the control group had a history of open angle glaucoma. The patient characters for both groups are shown in Table 1.

**Table 1:**
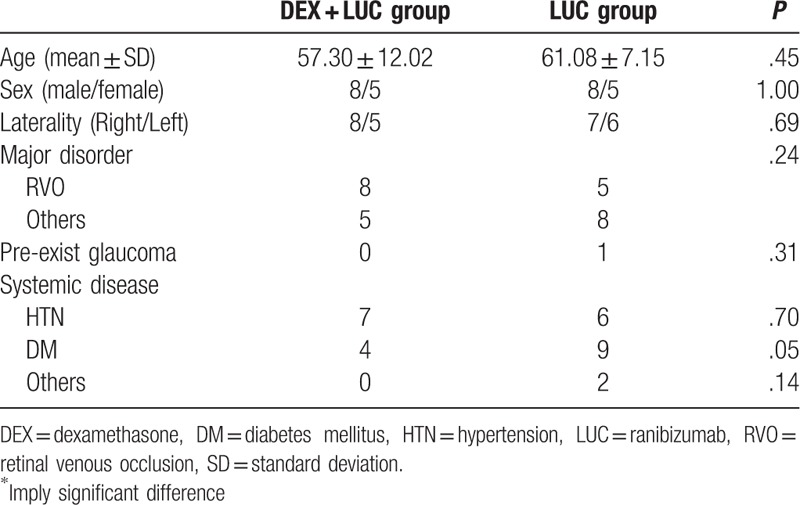
Patient characters.

**Figure 1:**
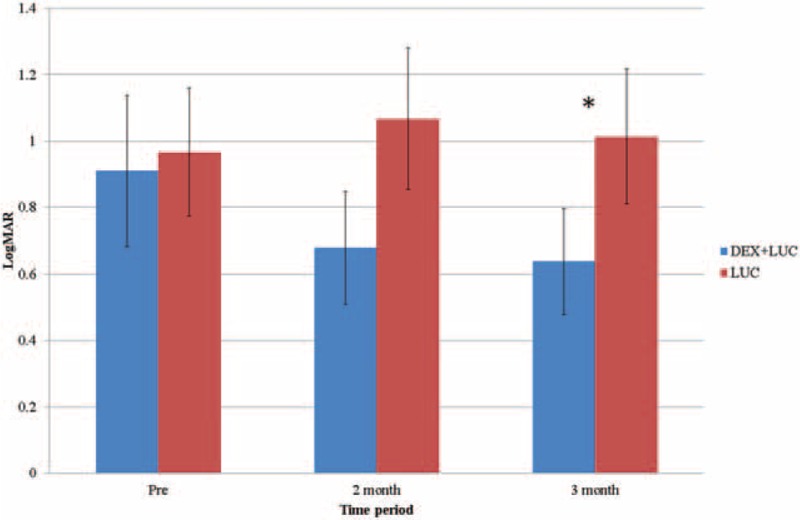
Comparison of the best-corrected visual acuity between the study and control groups. DEX+LUC = the study group with dexamethasone and ranibizumab, LUC = the control group with ranibizumab. ^∗^Indicates significant differences.

As indicated by LogMAR, the mean BCVA before treatment was 0.91 ± 0.61 (mean ± SD) in the study group and 0.96 ± 0.67 in the control group, without any significant between-group difference (*P* = .43). Two months after the concurrent injections, the BCVA improved to 0.68 ± 0.57 and further improved to 0.64 ± 0.59 3 months later with significant recovery (*P* = .04). No significant improvement was observed in the control group. The related data for the BCVA is shown in Table 2, and a between-group comparison of the BCVA is shown in Figure 1.

**Table 2:**
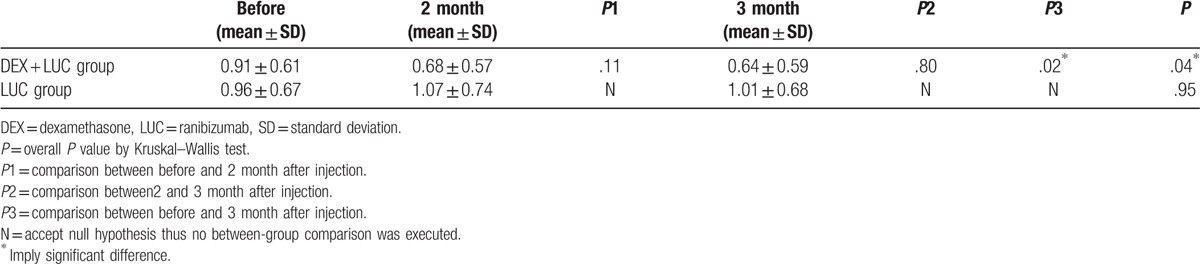
Visual performance after DEX implant by Log MAR.

**Figure 2:**
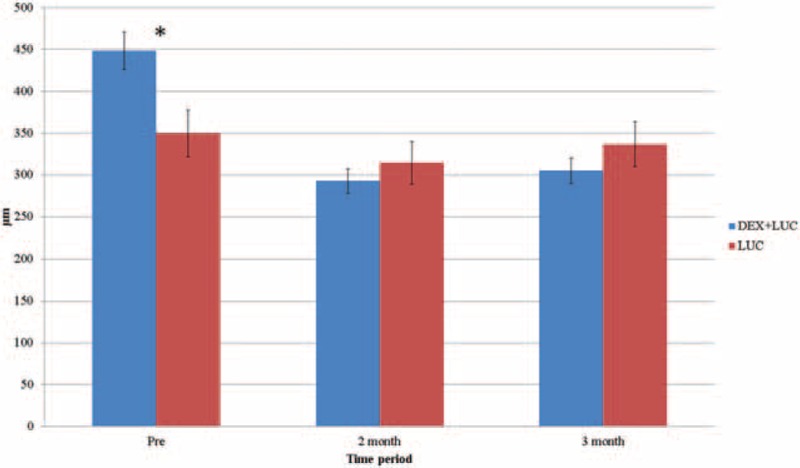
Comparison of central macular thickness between the study and control groups. DEX+LUC = the study group with dexamethasone and ranibizumab, LUC = the control group with ranibizumab. ^∗^Indicates significant differences.

Preoperatively, the mean CMT was 448.43 ± 143.07 μm in the study group, which was higher than that in the control group (349.40 ± 99.95 μm, *P* = .04). After the combined therapy, the CMT decreased to 292.62 ± 88.53 μm and stabilized at 305.18 ± 74.24 μm at 3 months postoperative with significant improvement (*P* < .01). No significant retardation of the CMT was observed in the control group. CMT changes are shown in Table 3, and the between-group CMT comparison is shown in Figure 2.

**Table 3:**
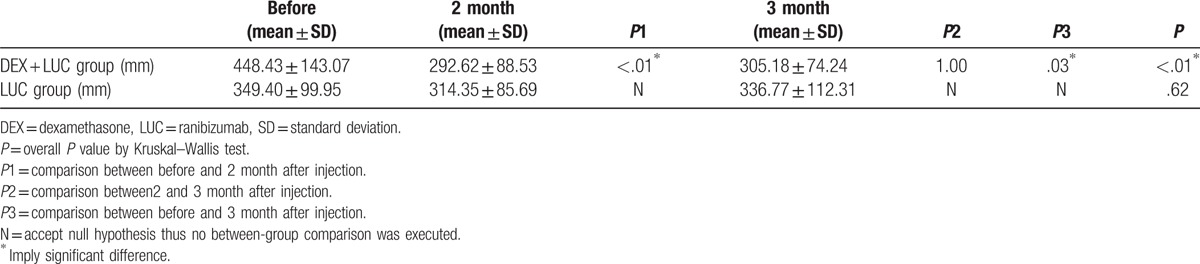
Central macular thickness after DEX implant.

**Figure 3:**
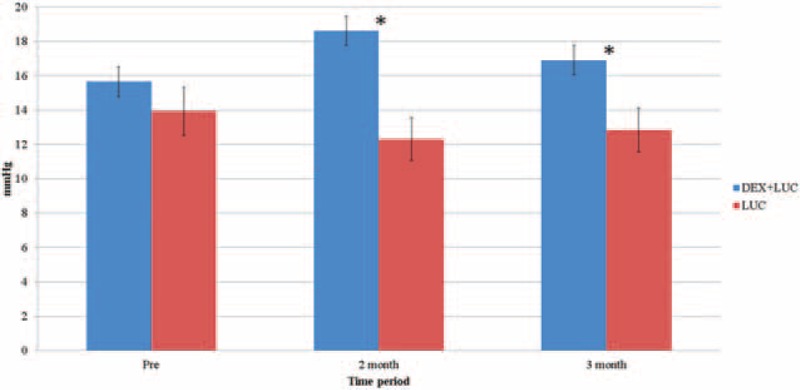
Comparison of intraocular pressure between the study and control groups. DEX+LUC = the study group with dexamethasone and ranibizumab, LUC = the control group with ranibizumab. ^∗^Indicates significant differences.

The preoperative IOP in the study group showed a mean value of 15.65 ± 2.93 mmHg, similar to the 13.92 ± 3.01 mmHg in the control group (*P* = .10). Two months after the injections, the mean IOP increased to 18.62 ± 4.06 mmHg and then dropped to 16.90 ± 2.57 mmHg 3 month after the treatment, while no significant elevation was found during the whole study interval (*P* = .15). Concerning the SIOH, only 1 patient had an elevated IOP of 10 mmHg (7.7%, at 2 postoperative months), which decreased 3 months after the surgery. No single IOP measurement of more than 30 mmHg was obtained. Five patients in the study group received medical treatment (38.5%), with travoprost in 3 patients and brimonidine in 2 patients. No patients required surgical intervention to reduce their refractory SIOH. Neither significant fluctuations of IOP nor any OHT episodes were observed in the control group. The IOP fluctuation is shown in Table 4, and the between-group comparison of the IOP is shown in Figure 3.

**Table 4:**
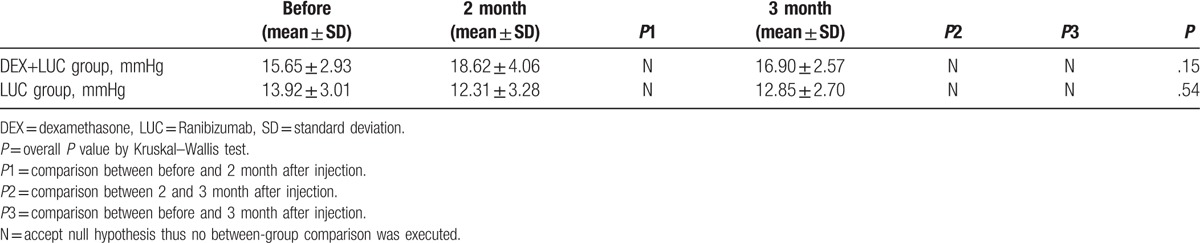
Intraocular pressure fluctuation after DEX implant.

Postoperative transient ocular pain was observed in all patients in the study and the control groups; however, the pain spontaneously subsided. Two patients with recurrent vitreous hemorrhage were recorded in each group. No new onsets of neovascular glaucoma were observed in patients, including 1 patient with central retinal venous occlusion and preexisting open angle glaucoma in the control group. Other severe complications, such as recurrent retinal detachment, vitreous leakage, suprachoroidal hemorrhage, wound rupture or prominent cataract formation, were not observed.

## 4 Discussion

In this study, the efficiency of concurrent DEX and ranibizumab injections was demonstrated through the significant improvement in the BCVA and CMT patients compared to the control group. In addition, the safety of DEX was also guaranteed, without significant IOP elevation in the whole study group, a result which has been suggested as a possibility in previous studies.^[12,17]^

The probably pathophysiology of SIOH is the modulation of steroids on the trabecular meshwork (TM) by increasing deposits at TM and suppressing the phagocytic effect in TM.^[3,22–28]^ Recently, numerous recent studies have evaluated the degree and management for the postoperative SIOH induced by DEX.^[12–19]^ Concerning the elevation of IOP, only 1 patient (7.7%) in our study experienced an IOP elevation of 10 mmHg from baseline, and no IOP measurements of more than 30 mmHg were recorded. The preliminary management revealed a much safer outcome compared to other studies with the percentage of SIOH ranged from 14.9% to 32.8%).^[12–17,19]^ The highest IOP ever measured in this study was 28.5 mmHg in the same patient whose IOP had increased 10 mmHg from baseline. A greater volume with the ranibizumab injection should induce a higher degree of SIOH theoretically, and previous studies have also revealed an elevated IOP after anti-angiogenic agent injection.^[29]^ Still, there are several possible explanations for our successful IOP control in which no significant IOP elevation was revealed in the study group. First, no patients with preexisting glaucoma, which is a prominent risk factors for SIOH,^[30,31]^ were enrolled in our study. Second, the mean age in our study group was 57.30 ± 12.02 years, younger than the 65.80 ± 12.90 and 71.90 ± 11.00 years in the other 2 studies,^[13,15]^ which may lead to better outcomes.^[32]^ In addition, anti-angiogenic agent may elevate IOP transiently, a finding that aligned with those of previous studies,^[33]^ and ranibizumab is not a toxic agent to the TM, which can decrease aqueous inflammatory cytokine, likely following OHT.^[34,35]^ Although all our patients received a single injection rather than multiple injections, this fact may be a minor reason for the fair IOP control because no significant difference in IOP has been observed between single and multiple administrations of DEX.^[12]^

For anti-glaucoma medications, prostaglandin analogues are the most effective agents, demonstrating a decrease of 28.00% in SIOH patients.^[36]^ In some advanced cases, destructive cyclophotocoagulation may be performed to diminish the progressive IOP elevation.^[8]^ In previous studies, the majority of patients with SIOH have been treated with medication successfully, while a small number of patients up to 3.2% still required surgical interventions.^[12–17,19]^ However, only topical antiglaucomatous agents were used for patients who experienced SIOH in this study. Even in the patient with the highest IOP, the IOP was reduced to 21.7 mmHg in the last follow-up visit after the administration of travoprost. The close postoperative follow-up in our institution, with a frequency of 4 to 5 times in the first 2 months, may help to recognize early SIOH and slow its progression before deterioration.

The effect of DEX on CMT retardation has been illustrated in previous studies.^[13,15,16,37,38]^ In this study, a significant improvement was observed in the reduction of CMT 2 months postoperatively, and the value stabilized at 3 months postoperatively in the study group, while no improvement was observed in the control group. DEX is an anti-inflammatory and anti-edematous agent that is theoretically stronger than anti-angiogenic agents concerning the treatment of ME,^[37]^ and many patients in control group were diagnosed with diabetic macular edema which is unresponsive to anti-angiogenic agent but can be retarded by DEX.^[39]^ Therefore, it is reasonable for CMT to achieve better recovery in the study group. Furthermore, the study group had a worse initial mean CMT value (448.43 ± 143.07 μm) compared to the control group (349.40 ± 99.95 μm, *P* = .04), while a previous study with similar outcomes reported identical preoperative CMT values between the DEX and control groups.^[37]^ Our findings suggest that the combined therapy may reduce CMT more effectively than anti-angiogenic agent, even for patients in worse conditions.

Another efficiency index, the BCVA, also improved in the study group, a result that correlated to the results of previous studies.^[13,15,16,37]^ In the current study, the BCVA was significantly improved in the study group at 3 postoperative months (*P* = .04), while no significant improvement was observed in the control group. Considering the visual acuity of 20/40 as a threshold, the ratio of patients to reach a BCVA above 20/40 in the final visit was 46.15% in the study group, which was higher than the 23.08% in the control group. Two patients in the study group even achieved an excellent BCVA of 20/20. This outcome conflicts with a previous report in which ranibizumab showed efficiency for BCVA recovery.^[40]^ However, many patients in our study were diagnosed with RVO, and in some patients with RVO, although BCVA cannot be improved by anti-angiogenic agents, it can be treated by DEX,^[41]^ implying that DEX may be a more universal choice for patients with ME for the recovery of BCVA.

Complications, such as cataract formation, conjunctival hemorrhage, DEX dislocation, eye pain, wound leakage, vitreous hemorrhage, intraocular infection, vitreomacular traction syndrome and iridocyclitis, have been reported after DEX implantation.^[6,42,43]^ The most prominent and persistent complication in this study was recurrent vitreous hemorrhage, which occurred in 2 patients in both the study and control groups. However, these 4 individuals had preexisting vitreous hemorrhage, thus, it is less likely that the complication had been induced by DEX or ranibizumab. Mild ocular pain was reported by all patients; however, it subsided in few days after the initial analgesic prescription. Cataract formation is a common complication after DEX implantation, and it has been reported in many studies.^[12,13,15,16,37]^ Interestingly, when using a silt-lamp biomicroscope, no cataracts were found in our study population. The short follow-up period of our study is the major reason for the absence of cataract formation, and some of our patients had already undergone cataract surgery before the combined therapy.

There are several limitations in our study. First, the small numbers of patients, with only 13 patients in each group, would diminish the statistical power of the study with a 22% chance of type II error. Second, the retrospective nature could limit the consistency, and some data, including underlying diseases, may be incomplete. Finally, the concurrent ranibizumab injection in the study group might influence the outcomes compared to previous studies.

In conclusion, concurrent DEX and ranibizumab injections are effective for treating ME, and their safety in terms of IOP fluctuation is guaranteed. Moderately severe and transient IOP elevation may occur in the combined procedure; however, it can be control through medical treatment with more convenience for patients compared to traditional methods. Future large-scale study that include different anti-angiogenic agents, such as aflibercept, are necessary to evaluate the long-term effect and safety of this combined treatment.
